# Therapeutic Tips

**Published:** 1907-06

**Authors:** E. S. M’Kee

**Affiliations:** Cincinnati


### Therapeutic Tips.

### RHINITIS IN CHILDREN, INCLUDING RECURRENT CORYZA DUE TO INTESTINAL INTOXICATION.

BY E. S. M'KEE, M. D., CINCINNATI.

Fisher (*N. Y. Medical Journal*) described what he terms intestinal rhinitis, a form caused by intestinal indigestion. It occurs frequently in dyspeptic children with residual and intestinal stagnation. The vaso-motor nerves supplying the mucosa of the nose, when affected by intestinal toxine, produce vaso-motor disturbances, one of which is recurrent coryza. Climatic conditions are naturally a minor factor in such conditions. When the intestine contains stagnant fecal matter, a general autointoxication results, frequently ending in rhinitis. These attacks last but two or three days. They recur unless a general cleansing of the gastro-intestinal tract is given at least as often as once a month. When recurrent rhinitis is seen in dyspeptic children, then the urine invariably contains indican. Not only is the presence of indican an important aid in eliciting the etiological factor in this type of recurrent rhinitis, but the presence of indican assumes a very important rôle in determining the proper therapeutic measures to be pursued. From what has just been said, we can easily see that if indican exists, associated with stagnant feces (chronic constipation), then the treatment is one of distinct elimination. It is seen that the diagnosis is strengthened by the presence of either large or small quantities of indican in the urine, and the stronger the blue reaction of indican the larger the amount present. Hence the point would be to keep up the eliminative treatment till the urine shows less and less or only the normal traces of indican. Meat should be prohibited. Saline cathartics should be given; sodium phosphate should be given till it produces liquid stools. Water should be given freely, and fruit may be given in abundance. Milk, buttermilk, cereals and butter should be prescribed. The author regards it as irrational to use the familiar oil sprays and powders. The proper plan is to seek out the cause of the rhinitis and remove it. We should remember that rhinitis may usher in meningitis, various eruptive fevers, diphtheria, influenza and specific diseases.

### TO CURE THE ITCH IN NINETY MINUTES.

Saboureaud (*Bulletin Generale de Therapeutique*) says this is attainable by the following process: The patient is rubbed for half an hour with soft soap, which is followed by an alkaline bath for a half an hour. Then the entire body is rubbed with the following: Oil verbena, gum tragacanth of each one part, precipitated sulphur 100 parts, glycerine 200 parts. Mix well. A final bath is then taken lasting from fifteen to twenty minutes. The clothing and body linen must be disinfected. In the two weeks following this treatment four baths of starch water are given. In case there should be cutaneous irritation, local applications of zinc ointment should be made.

### PAINLESS MERCURY INJECTIONS.

Mayer (*Deutsch Med. Wochenscrift*) reports from Lassars clinic and experience with 900 injections of a combination of mercuric cyanide acoin and boracic acid. Fifteen times using 1.6 per cent solution pain was complained of, forty-three times 4.7 per cent solutions uncomfortable sensations were caused. All other injections caused no pain or discomfort. No other undesirable results ensued. The author believes this new solution to be a distinct improvement on all others which he has used. Following is his formula: Hydrargyri cyanidi 1. Dissolve with gentle heat in 1 per cent of boracic acid in distilled water 30. 2. Aconi (von Heyden). Dissolve in 1 per cent of boracic acid in distilled water 70. M. Dispense in opaque bottles. Sig. One or 2 cc. as an injection.

### FOR GONORRHEAL RHEUMATISM.

℞ Acidi salicylic dr. i or 4, mentholis gr. xv or 1, Guiacaolis dr. ss or 2, alcoholis oz. i or 30. M. Sig.: Paint over the affected areas and cover the parts with oiled silk.—*Merck's Archives.*

PERINEURAL SALINE INJECTIONS IN SCIATICA.

Grossman (*Wiener Klinische Wochenschrift,* October 18, 1906) reviews the history of this method of treating sciatica and then describes his experiences. He treated fifteen patients with severe and chronic sciatica, and all were relieved at once of the pain. This striking result he found not always permanent. The pain returns sometimes in a milder form but is then amenable to such measures as superheated air, mud baths, hot packs, leeches, and the like. No by-effects were noted in any case. The prompt action in banishing the pain, he declares, certainly justifies the use of this simple and harmless measure. He used a 0.6 per cent salt solution, injecting from 50 to 100 grammes. The patient complains of pain in the limb and tingling as the needle approaches the nerve. In a few instances the injection was repeated. All but one of his patients were cured or materially improved.

Sciatica is discussed by Granger (*Journal of Physical Therapy*). He considers that on account of its anatomical course the sciatic nerve is peculiarly liable to injury from trauma and exposure to cold, hence one of the most common as well as stubborn diseases. After having carefully settled the etiology, we must determine in what part of the nerve the lesion is situated. According to Snow, in 90 per cent this is at or near the sciatic notch. Flat foot should be looked out for and if present corrected by plates and proper fitting shoes ordered. At the Boston dispensary, where the author does his work, a large number of cases are referred to his clinic and are treated on the histories they bring with them. In case success does not follow, the cases are gone over carefully and an effort made to determine the cause of the failure. This, if the case is carefully studied, is frequently found and the case cured. This is often found to be osteo-arthritis of the lumbar region of the spine, which condition is discovered by having the patient stand erect, arms hanging down the sides, the lower portion of the back bared, and bend forward, backward, to the right, to the left, first seeing if the spine is flexible, and next if there is limitation of motion in any direction, or spasm of the muscles. The urine should be examined for sugar. The nerve reflexes should be gone over to rule out any organic diseases of the spinal cord. Hip-joint disease must not be forgotten. We must remember that a loaded rectum or hemorrhoids can cause sciatica from pressure, as well as malignant cause, which is also a causative factor. In chronic cases the author used the X-ray in order to "produce absorption of any old exudate or scar tissue pressing on the nerve." He had the patient take quinine, four grain at intervals of an hour, two doses, with the object of producing fluorescence of the blood, as recommended by Morton. The patient lies on the table, back up, the notch and the course of the nerve exposed. The genitals and the other leg were well covered with lead, and with a tube which will readily expose the small bones of the wrist at a distance of eighteen inches from the tube; an exposure of ten minutes is given at a distance of eight inches. The lower part of the nerve is rayed for eight minutes more, and this is followed by a high-frequency treatment of ten minutes more, or a light treatment of fifteen, as thereby I feel sure that the liability of producing a burn is reduced to the minimum. From the high-frequency attachment of the static machine or coil, or from the high-frequency coil itself, the high-frequency current applied by means of a glass vacuum tube has produced good results. Similarly, cases have been relieved by the various high candle lamps on the market, but more especially by the minim blue light, for with the blue light we get much deeper penetration than from any other incandescent spectrum alone, regardless of the amperage consumed by the filament. With the blue light he uses as much heat as the patient can stand for at least a half hour. The exposure is made over the bare skin, and when it becomes too hot I rub in some of the many preparations containing iodine or the iodides containing an oleaginous base, thus getting not only the blue end of the spectrum but also the iodine effect plus that of hot oil.

